# From superior contrast to super resolution label free optical microscopy

**DOI:** 10.1038/s44303-024-00064-w

**Published:** 2025-01-13

**Authors:** Nikhil Jayakumar, Balpreet Singh Ahluwalia

**Affiliations:** 1 Department of Physics and Technology, UiT, The Arctic University of Norway, Tromsø, Norway; 2 Department of Clinical Science, Intervention and Technology, Karolinska Insitute, Stockholm, Sweden

**Keywords:** Optics and photonics, Physics

## Abstract

Label-free optical microscopy utilizes the information encoded in light scattered off unlabeled particles to generate the images. This review article starts off with a discussion on how this light matter interaction gives rise to the issues of poor-contrast and diffraction-limited spatial resolution. Then, this article reviews the various far-field label-free optical microscopy techniques that have been developed, with an emphasis on the physical mechanisms behind the image formation processes in such techniques. Thus the article aims to elucidate the various state-of-the-art label-free techniques and their current applications.

## Introduction

An optical microscope employs visible light to unravel the mysteries of the microscopic world. This minimally invasive tool is used by labs across the world, with applications ranging from embryonic imaging, and cell biology to drug research^1–6^. Since its invention, the major challenges have arguably been diffraction-limited spatial resolution, poor contrast and multiple scattering associated with thick specimens. However, courtesy of fluorescence microscopy^7^, the challenges of poor contrast and limited spatial resolution have been considerably mitigated which subsequently led to the recognition of the field of fluorescence-based super-resolution microscopy^8^ with the 2014 Nobel Prize in Chemistry. In these fluorescence-based techniques, an image of the locations of the exogenous fluorescent molecules is generated. By exploiting these molecules’ molecular specificity, they achieve better contrast images. Then to circumvent the spatial resolution limit the photo-physics of these exogenous molecules is utilized via a spatially non-uniform illumination or/and with a non-linear photo-response from the molecules. This helps capture different far-field diffraction-limited images that finally help generate a super-resolved image, albeit at the cost of reduced temporal resolution. Though these techniques are used extensively by labs, limited photon budget, photo-toxicity, photo-bleaching, spectral proximity of different fluorescent labels in the sample etc mandate label-free techniques with similar capabilities. That is the onus is on developing label-free microscopes with superior contrast and superior resolution in space and time, to visualize the dynamics of the microscopic world. Why mitigating these problems has been more challenging in far-field label-free microscopy as opposed to fluorescence microscopy may be attributed to the “extra handle”offered by the exogenous fluorescent molecules. Or simply put, the nanosize and photo-physics of the plethora of bio-compatible exogenous fluorescent molecules at visible wavelengths offer ingenious ways of circumventing the far-field Abbe limit as opposed to label-free microscopy. Despite these challenges, there has been considerable progress in the field of high-contrast and high-resolution label-free microscopy. This field of label-free microscopy is ever-expanding and a comprehensive review may be found in ref. ^9–11^. In this article, a few of the commonly employed far-field label-free optical microscopy techniques are discussed. The discussion is restricted to light-matter interaction techniques in the visible domain. The article is formulated into two main sections, discussing various ways to achieve superior contrast and superior resolution. The physical mechanism behind the image formation processes involved is discussed in detail, with an emphasis on the importance of coherence of light. This emphasis helps to shed light on how and why manipulation of coherence of light is helpful in imaging with better contrast and resolution and thereby, also aid in resolving confusion regarding the usage of terminologies such as high-resolution and super-resolution in the context of label-free microscopy.

## Terminologies

Typically in optical microscopy, the signal-to-noise ratio (SNR) is calculated to quantify the image contrast which indicates how well different regions of interest in the image can be differentiated. SNR may be calculated as the ratio of average $\overline{x}$ to standard deviation *σ* of the pixel values in a region. This standard deviation may be either computed spatially over the pixels in the region of interest or computed temporally for each pixel, and its origin is attributed to noise in the system.

1 $$SNR=\frac{\overline{x}}{\sigma }$$

A SNR value of 1, Eq. (1), implies that the structure of interest is not discernible from the noise. Another metric commonly employed for quantifying contrast to discern two points of interest is the contrast-to-noise ratio (CNR). For e.g., to quantify contrast between two points ’A’ and ’B’, CNR may be computed as given by Eq. (2)^12^.

2 $$CNR=\frac{| {x}_{A}-{x}_{B}| }{\sigma }$$

Therefore, a high SNR is important as it limits detection of weakly scattering specimens, localization precision, and spatial resolution of the final image.

**Fig. 1:**
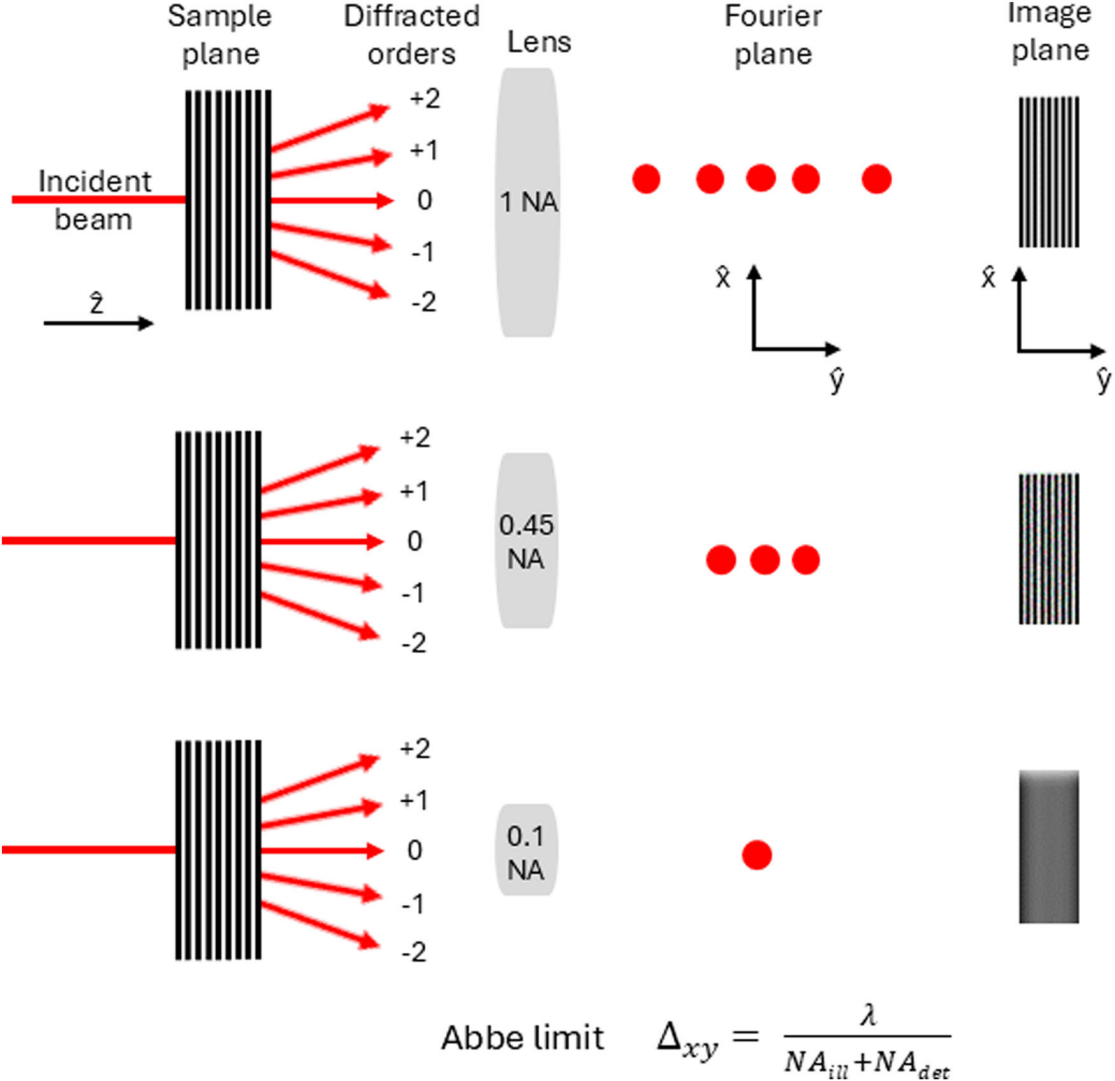
Schematic explanation of the Abbe diffraction-limit. An incident light beam along $\hat{z}$-axis, shown in red, illuminates a sample. The light undergoes diffraction at the sample plane and the diffracted orders for this particular sample are shown by the red arrows. Now, to acquire an image of this sample, a lens is used in the far-field to capture the light emanating from the sample. The effect of the light-gathering capacity of such a lens is quantified in terms of the numerical aperture (NA) and the influence of various NA is shown. In the first case, NA = 1, all the diffracted orders off the sample are collected by the lens. The transmitted field at the second focal-plane of the lens, i.e., the transverse $\hat{x}$-$\hat{y}$ plane, is equivalent to Fourier transform of the field at the first focal plane and therefore, is referred to as the Fourier plane. The Fourier plane shows the admitted Fourier components of the sample into the system. And the image formed by superimposing these Fourier components in the far-field $\hat{x}$-$\hat{y}$ image plane, which is typically achieved using a tube lens in a microscope, is shown. In the second case, NA = 0.45, the ± 2 diffraction orders are beyond the collection angle of the lens and are not admitted into the system. An image of the sample is still generated at the image plane albeit, with a poorer resolution in comparison with the system with NA = 1. That is the finer details of the specimen, ±2 orders, are lost upon propagation through the system. Only the zero and ±1 diffracted orders are admitted into the system, as seen in the Fourier plane, and these orders superimpose at the image plane to generate the final image. In the third case, NA = 0.1, only the zero order is admitted into the system and the image plane exhibits no modulation in intensity. However, by tilting the illumination beam, higher-orders off the sample may be admitted into the system, thereby capturing the finer details of the specimen which can give rise to modulations in intensity at the image plane. Based on these findings, Abbe concluded based that image formation is an interference effect of a diffraction phenomenon and the best achievable resolution is ${\Delta }_{xy}=\frac{{\lambda }_{det}}{N{A}_{ill}+N{A}_{det}}$.

The spatial resolution of a microscope is defined as the limiting distance between two particles such that the particles appear just resolved in the image generated by the microscope. Though different criteria such as the Rayleigh limit, Sparrow limit etc are used in the microscopy community, the Abbe limit derived from physical principles by Ernst Abbe in 1870, is accepted as the best resolution that can be achieved in principle. But recent studies by Prof. Evgenii Narimanov, where the image formation process is treated within the framework of information theory suggests that an even higher resolution can be achieved if a priori information of the sample is known or if the SNR is good^13^. However, the Abbe limit is commonly used within the community to quantify the best achievable resolution. The limit implies that using an on-axis illumination, the best achievable transversal resolution Δ_*xy*_ is *λ*_*det*_/*NA*_*det*_, where *λ* is the wavelength of the detected light and *NA*_*det*_ is the numerical aperture of the detection system. For off-axis illumination of coherently scattering specimens, the Abbe limit is modified as

3 $${\Delta }_{xy}=\frac{{\lambda }_{det}}{N{A}_{ill}+N{A}_{det}}$$

where *NA*_*ill*_ is the numerical aperture of the illumination system. This concept is illustrated schematically in Fig. 1. The Abbe limit implies that at visible wavelengths (≈400–700 nm) the smallest separations that a far-field optical microscope can resolve is ~200 nm. Now Eq. (3) gets modified for incoherent imaging as the illumination phases are lost. That is, for example in fluorescence microscopy, the fluorophores emit incoherently and hence, the best achievable resolution is *λ*_*det*_/2*NA*_*det*_. Thus, the Abbe limit provides a benchmark to quantify the spatial resolution limit and thereby, also classify the system into various categories based on this limit. The imaging system may then be classified into diffraction-limited or standard limit, extended resolution beyond the diffraction-limit, and super-resolution imaging systems^14,15^.

## Challenges

The physical mechanism behind the reason for poor contrast and diffraction-limited resolution is worth elucidating to understand the challenges and in developing solutions for these challenges. In optical microscopy, visible light is used to illuminate the sample under study. This illuminating field gets scattered by the sample, i.e., light undergoes diffraction. The evolution of this field in space may be understood by invoking the scalar Helmholtz equation which is derived from the Maxwell equations. Then the equations for the scattered fields helps to understand the physical mechanism behind the challenges of poor contrast and finite spatial resolution. For brevity, a monochromatic scalar scattered field ${u}_{s}(\overrightarrow{r},\omega )$ is considered to elucidate the concepts.

### Poor contrast

In experiments it is observed that imaging of thin biological specimens using visible light is challenging. That is, it is difficult to discern the features of interest from the background. This can be understood by expressing the scalar inhomogeneous wave equation for scattered field as demonstrated by Prof. Emil Wolf in ref. ^16^. The temporal dependence of the fields is omitted for brevity.

4 $$\left[{\nabla }^{2}+{k}_{m}^{2}\right]{u}_{s}(\overrightarrow{r})=-f(\overrightarrow{r})u(\overrightarrow{r})$$

where, *k*_*m*_ = 2*πn*_*m*_/*λ* is the wave number, *λ* is the free space wavelength, ${u}_{s}(\overrightarrow{r})$ is the scattered field, $f(\overrightarrow{r})={k}_{m}^{2}\left[\frac{{n}^{2}(\overrightarrow{r})}{{n}_{m}^{2}}-1\right]$ is the scattering potential, $u(\overrightarrow{r})$ is the sum of incident and scattered fields, $n(\overrightarrow{r})$ is the refractive index of the sample and *n*_*m*_ is the refractive index of the surrounding medium. The right-hand side of Eq. (4) accounts for the inhomogeneity and acts as the source for the scattered field. At visible wavelengths for thin biological specimens, $n(\overrightarrow{r})\approx {n}_{m}$. It implies that the strength of the scattered field, i.e., the response of the biological sample to the incident field, is weak in comparison to the incident field. More insights about the scattered field can be gained from Eq. (4) via a simplification, i.e., invoking the Born approximation^17^. This approximation is valid while considering particles whose radius, ‘a’, is smaller than the wavelength of the incident field^18^, $\frac{2\pi a}{\lambda } < < 1$. The approximation leads to the differential scattering cross-section of particle, defined as the square of the modulus of the scattering potential, to show 1/*λ*^4^ and *a*^6^ dependence. That is, the scattered intensity depends strongly on both size of the scattering particle and wavelength of the incident field. For e.g., a particle of radius 10 nm illuminated with blue light scatters ≈10^10^ times stronger than a 1 nm particle illuminated with red light of the same strength. The consequence is that imaging of thin biological specimens using visible light is challenging.

### Loss in spatial resolution

Next, the issue of finite spatial resolution is analyzed. The evolution of the scattered field along say *z*-axis can be calculated using the scalar Helmholtz equation as given in Eq. (5).

5 $${\nabla }^{2}{u}_{s}(\overrightarrow{r},\omega )+{k}_{m}^{2}(\omega ){u}_{s}(\overrightarrow{r},\omega )=0$$

Note that Eq. (4) can be arrived at by rearranging the terms in Eq. (5) by explicitly showing the source term on the right hand side. To solve Eq. (5), a spatial Fourier transform helps to convert the differential equation into an algebraic equation. A physically valid solution of this equation for z > 0 is ${\tilde{U}}_{s}(\overrightarrow{k},\omega )=\tilde{U}(\alpha ,\beta ;z)exp[i\gamma (\alpha ,\beta )z]$. Here *α* = *k*_*x*_, *β* = *k*_*y*_ and *γ* = *k*_*z*_ are the spatial frequencies and satisfy the dispersion relation, ${k}_{m}^{2}={\alpha }^{2}+{\beta }^{2}+{\gamma }^{2}=\frac{{\omega }^{2}}{{c}^{2}}{\epsilon }_{m}(\omega )$. ${k}_{m}=\frac{\omega }{c}{n}_{m}(\omega )$ is the wavenumber in a medium of permittivity ${\epsilon }_{m}(\omega )=\sqrt{{n}_{m}(\omega )}$, *n*_*m*_(*ω*) is the complex refractive index associated with the sample and the magnetic permeability is assumed to be one which is typically the case at optical frequencies. Due to the constraint imposed by the dispersion relation, for subwavelength features *γ* becomes imaginary, i.e., when *α*^2^ + *β*^2^ > *k*^2^. Hence, depending on the value of *γ*, homogeneous or evanescent waves constitute the scattered field ${u}_{s}(\overrightarrow{r},\omega )$. That is, structural information of the sample smaller than the wavelength of light is encoded in the higher spatial frequency components of the scattered field and they propagate with an exponentially decaying amplitude with distance, $\tilde{U}(\alpha ,\beta )exp(-\gamma z)$. That is, free space propagation performs low-pass filtering on the Fourier components of the scattered field.

This scattered field is then intercepted by the detection microscope objective placed in the far-field of the sample. Now the the finite size of the front focal plane of the detection objective restricts the collection of the low-passed sample information. As a result, only certain Fourier components of the propagated field are admitted into the system and this finite acceptance angle is quantified as the numerical aperture (NA) of the detection system. The NA thus performs an additional low-pass filtering on the propagated sample information in the far-field, see Fig. 1 for a schematic illustration. In addition to low-pass filtering by propagation and NA of the imaging system, the pixel size of the digital camera employed for recording the images, imposes a constraint on the magnification of the system. This constraint arises due to the Nyquist sampling criteria, which mandates that the smallest resolvable distance at the sample plane must be magnified by the imaging system to span at least two pixels of the imaging sensor. Such an imaging system in principle will be able to satisfy the Abbe limit, Eq. (3).

## Methods to combat poor contrast

In this section, a few of the commonly employed label-free microscopy techniques to combat the issue of poor contrast is discussed.

### Category 1: darkfield microscopy

The main result in Section “Challenges” with regards to poor contrast is that the scattered field gets buried in the presence of an overwhelming illumination field. Therefore, this section describes techniques that allow to cut out the illumination field and allow only scattered light off the sample to reach the camera. A comprehensive review on this topic may be found in ref. ^19^.

#### Ultramicroscope

An obvious solution to the issue of poor contrast is to allow only the scattered fields to reach the detector. This was first experimentally realized by Richard Adolf Zsigmondy by resorting to an orthogonal illumination and detection scheme and the developed system was called an ultramicroscope. The ultramicroscope enabled the study of the composition of colloidal solutions. The significance of this work was recognized with the Nobel Prize in Chemistry in 1925 to Prof. Zsigmondy^20^. Ever since its invention, several similar techniques have emerged that use the concept of geometric separation between the illumination and detection paths^21^. Such techniques which allow only the scattered fields to reach the camera are commonly referred to as darkfield techniques. The name can be attributed to the fact that the structures of interest are visible as bright regions on a dark background.

Now for 3D imaging, cylindrical lenses are used to generate light sheets for decoupled illumination and detection. A better focusing along the axial direction helps achieve better sectioning, albeit at the cost of poor uniformity over the detection objectives field-of-view (FoV). Also, 3D specimens such as organs can have large variations in refractive index which can cause multiple scattering of the illuminating field. This problem can be mitigated via optical clearing of tissue sections which was first developed by Spalteholz in 1914^22^. Nowadays, the concepts of light sheet microscopy and tissue clearing are combined to perform label-free 3D microscopy and is gaining attention for various applications^23,24^.

#### Prism-based darkfield microscopy

In 1956 E.J. Ambrose invoked the concept of total internal reflection (TIR) based illumination scheme to image only the scattered fields^25^. The basic concept behind TIR microscopy is that when waves undergo TIR at an interface, say a glass-water interface, an evanescent field is simultaneously generated in the water. This field exponentially decays in amplitude to satisfy the boundary conditions. Such a limited penetration depth allows only the sample’s regions near the glass-water interface to scatter light significantly into the far field. Thus, TIR illumination helps generate high-contrast images of regions of only structures which are within the penetration depth of the field.

In the first setup demonstrated in 1956^25^, light from an intense mercury arm lamp was passed through a slit and made to undergo TIR at the glass-water interface using a prism. The evanescent field generated inside the less dense medium enabled the study of the contacts made by cells immersed in water on the glass prism. Nowadays surface plasmon resonance enables even shorter penetration depths of the evanescent fields and can be generated using Otto, Kretschman configurations^26^. Surface plasmons are the eigen waves supported at a metal/dielectric interface, i.e., they are the collective excitation of the conduction electrons of the metal. The penetration depth of a few hundred nanometers of such waves enables label-free real-time analysis of biomolecule interactions^27^ and imaging and quantification of single viruses^28^.

#### Objective-based TIR-darkfield microscopy

The basic concept is the same as prism-based TIR and was introduced by Axelrod^29^, initially for fluorescence microscopy. Light travelling in a medium with refractive index *n*_1_ encounters a medium of refractive index *n*_2_ such that *n*_1_ > *n*_2_. Using Fresnel equations for reflection and refraction, it is shown that if the angle of incidence at the interface is greater than the critical angle for the particular interface, *θ*_*c*_ = *sin*^−1^(*n*_2_/*n*_1_), the incident light undergoes TIR at the boundary.

Typically in experiments, a high NA objective is used to achieve supercritical angle of incidence. Then a focused off-axis light beam is made to strike the objective’s back focal plane. This ensures a collimated oblique beam at the front focal plane of the objective so that TIR is satisfied at the glass-liquid interface. These techniques have allowed the detection of 60 nm polystyrene beads^30^, gold nanoparticles^30–32^, single molecule binding kinetics^33^ etc. The detection sensitivity is shown to degrade depending on unwanted scattering arising from any index perturbations^34^. This technique is commonly employed by labs across the world for high-contrast diffraction-limited imaging. Recently, courtesy of convolutional neural networks label-free super-resolution in dark-field configuration has been demonstrated^35^, opening up new avenues for machine learning-assisted high contrast high-resolution label-free imaging.

#### Dielectric optical waveguide-based microscopy

Waveguide based dark-field microscopy is used extensively since the late 90’s in both fluorescence^36–40^ and label-free microscopy^41–44^ microscopy. This is attributed to scalable FoV imaging^45^, multi-wavelength TIR operation^46,47^, mature foundry techniques, higher peak intensities of the evanescent field^45^, uniform illumination^45^, complex beam shaping on a smaller footprint^37,43^ and miniaturization. Optical waveguides confines the diffraction of light and transmit optical power over longer distances. The light coupled into a high-index core material sandwiched in between lower-index cladding gets guided along the length of the waveguide via TIR. Simultaneously, an evanescent field is set up along the core-cladding interface along the entire length of the waveguide. The coupled optical power gets transmitted via the optical modes supported by the guiding structure. The optical modes can be understood as solutions to the wave equation obtained after invoking the appropriate boundary conditions for the particular waveguide geometry considered. For brevity, a film waveguide with a core refractive index *n*_1_ sandwiched between cladding with refractive index *n*_2_ is considered, such that *n*_1_ > *n*_2_. The transverse electric (TE) modes of this structure is as follows^48^.

6 $${E}_{y}(x)=\left\{\begin{array}{ll}A\cos \kappa x\quad &;| x| < -d/2;\\ Cexp(-\gamma | x| )\quad &;| x| > +d/2\end{array}\right.$$

In Eq. (6), ${\kappa }^{2}={k}_{0}^{2}{n}_{1}^{2}-{\beta }^{2}$, ${\gamma }^{2}={\beta }^{2}-{k}_{0}^{2}{n}_{2}^{2}$, *β* is the propagation constant, *d* is the core width and *k*_0_ = *ω*/*c* is the vacuum wave number. The guided modes in the core assume oscillatory behavior while in the cladding they assume an exponentially decaying behavior to satisfy the boundary conditions. Assume *d* = 4 μm, *n*_1_ = 1.503 and *n*_2_ = 1.500, the effective index of the guided mode is *n*_*eff*_ ≈ 1.5016. It lies between the core index and the cladding index which allows TIR. Hence, by suitable choice of core and cladding indices, the effective index of the guided modes can be changed which also changes the penetration depth 1/*γ* of the evanescent field.

What effect do these mode profiles or in general TIR-based illumination scheme have on imaging? This can be understood by calculating the net energy flow, i.e., the time-averaged Poynting vector along the detection arm is zero. This is because of the *π*/2 radians phase difference between the electric and magnetic fields along the detection axis^49^. It means that on average no power flows into the camera. This implies that the illuminating light does not propagate into the camera. But whenever there is an index perturbation at the core-cladding interface, i.e., the presence of a biological sample, the light gets scattered into the camera. Hence, a high-contrast image may be generated due to the absence of the overwhelming illumination light.

Now the illumination of the sample is via an evanescent wave, i.e., an oblique beam. It means that higher spatial frequencies of the sample get low-passed into the passband of the detection arm. This can be understood by invoking the first-order Born approximation, i.e., assume *u* ≈ *u*_*i*_ and taking the Fourier transform of Eq. (4). If the incident field is a plane wave, ${u}_{i}=exp(i{\overrightarrow{k}}_{\!i}.\overrightarrow{r})$ and *u*_*i*_ > *u*_*s*_, then the spatial frequency transmitted into the detection objective is $\tilde{F}$ ($\overrightarrow{k}$ - ${\overrightarrow{k}}_{\!i}$), where the magnitude of the illumination wave vector gets scaled up by the refractive index of the medium^43,50^. For e.g., the refractive index of Si_3_N_4_ and Ta_2_O_5_ at visible wavelengths is ≈2 and therefore, the magnitude of the wave vector is almost twice that of in free space. An even higher magnitude of the wave vector can be generated by exciting the surface plasmon waves, and the use of plasmons in microscopy is gaining popularity^51^. Based on the refractive index contrast between the core and the cladding, waveguides may be classified into high-index contast (HIC) and low-index contrast (LIC). Common examples for HIC waveguides include Si_3_N_4_ core on SiO_2_ substrate^43,46^, Ta_2_O_5_ on SiO_2_ substrate^47,52^, AlO_*x*_ core on SiO_2_/Si substrate^38,44,53^, GaP^39,54^. LIC waveguide design tries to index match the core-cladding layers, for e.g., SiO_2_ on CYTOP^42,55,56^. Typically in waveguide-based microscopy, the biological sample embedded in an appropriate medium is placed inside a ≈150 μm polydimethylsiloxane chamber on top of the higher index core material. Laser light is coupled into the waveguide via a coupling objective MO_1_ and using a detection objective MO_2_ the scattered light off the sample is relayed onto the camera^57^. This technique has enabled high-contrast imaging of biological cells^38,43^, sub-100 nm lipid vesicles^42^, protein binding to individual surface-bound lipids^58^ etc.

#### Opto-fluidic and hollow-core optical fibers

Tracking of nanoparticles over longer periods can be achieved by using opto-fluidic or hollow-core fibers. Opto-fluidic fibers can have a hollow cylinder of about 250 nm diameter in doped silica of about 3 μm which serves as the core. Here, the nanoparticles are confined to the hollow-cylinder and interacts with the leaky light from the guided light in the core. The hollow-cylinder serves as both light guidance and micro/nano fluidic channel enables confinement of the freely diffusing particles to detect diffusing 19 nm nanoparticles^59^. In another scheme, nanofluidic chips are used. Nanochannels that spans tens to a few hundred nanometers are used for restricting the motion of freely diffusing particles to within the focal volume of the detection objective. An oblique beam illuminates the sample flowing in the nanochannel and through the use of micromirrors, the reflected light off the chip is cut-off and only the scattered fields make it to the camera^60^.

Hollow-core fibers have also made their mark by imaging of even sub-10 nm diffusing particles^61^ and a recently developed technique is abbreivated FaNTA, which stands for Fiber-assisted Nanoparticle Tracking Analysis (NTA)^62^. The guidance mechanism can be explained as light suffering a near-unity reflection from the silica cladding into the hollow-core close to the grazing angles of incidence. The advantage over conventional NTA, as the name of the technique suggests, is the usage of hollow core geometry to encapsulate the analyte and the particle. Due to the confinement of the particle within the core, light interacts with the particle for longer periods of time allowing better sensitivity than NTA. Some of the other popular techniques include dynamic light scattering (DLS), particle tracking analysis etc. A review of these techniques is provided in ref. ^63^. It must be noted that the primary aim of these techniques have not been in imaging the morphology of these nanoparticles as their morphological features are beyond the resolving capabilities of the optical imaging system, but rather in detecting and characterizing the size distribution of the nanoparticles.

### Category 2: interferometric microscopy

Interference between light waves is used to improve resolution and sensitivity while imaging thick biological tissues using optical coherence tomography (OCT)^64^ or when imaging of single proteins via interferometric scattering microscopy (iSCAT)^65^. Conventional bright-field microscopy (BFM), phase contrast microscopy (PCM), differential interference contrast (DIC), quantitative phase microscopy (QPM), rotating optical coherence scattering microscopy (ROCS), iSCAT etc rely on interference between scattered and reference waves to generate optical images with better resolution and contrast. While PCM, DIC, QPM, ROCS are primarily used for imaging of biological cells with better contrast and resolution, the potential of iSCAT lies in improved detection sensitivity of even weakly scattering specimens like single proteins. In this section, these techniques are discussed elaborating the physical mechanism behind their working principle.

#### BFM

Abbe put forward the concept of image formation as an interference effect of a diffraction phenomenon. The underlying equation for image formation after omitting the temporal dependence can be described as follows.

7 $${I}_{det}(\overrightarrow{r})=| {u}_{s}(\overrightarrow{r}){| }^{2}+| {u}_{i}(\overrightarrow{r}){| }^{2}+2| {u}_{i}(\overrightarrow{r})| | {u}_{s}(\overrightarrow{r})| \cos (\Delta \phi (\overrightarrow{r}))$$

where ${I}_{det}(\overrightarrow{r})$ is the detected intensity at the camera plane, ${u}_{i}(\overrightarrow{r})$ and ${u}_{s}(\overrightarrow{r})$ are the complex-valued illumination and scattered fields overlapping at the camera plane, and $\Delta \phi (\overrightarrow{r})$ is the phase difference between the overlapping fields. For thin biological specimens at visible wavelengths, $\Delta \phi (\overrightarrow{r})$ is small and therefore, the interference term in Eq. (7), $\cos (\Delta \phi (\overrightarrow{r}))\,\approx 1$. This implies minimal modulation in intensity in the generated image^12^.

#### PCM

Frits Zernike was awarded the 1953 Nobel Prize in Physics for building upon Abbe’s image formation theory to develop PCM capable of visualizing weakly scattering specimens with improved contrast. The trick employed in PCM is the introduction an additional phase delay of *π*/2 rad between the illumination and scattered fields via a phase contrast filter. It means that the interference term in Eq. (7) can be rewritten as $\sin (\Delta \phi (\overrightarrow{r}))$. For weakly scattering specimens, this can be approximated as $\Delta \phi (\overrightarrow{r})$. That is, the intensity detected at the camera is a linear function of the phase difference between the overlapping fields and this allowed for improved image contrast^66^.

#### DIC

Initially invented by Francis Smith in 1947 and later modified by Georges Nomarski in 1952, DIC helps generate high-contrast images. Typically, a Wollaston prism is used to generate two light beams with a shear between them. The beams are orthogonally polarized and illuminate the sample under investigation. The two beams accumulate different phases upon propagation through the sample and are made to overlap via another Wollaston prism. The overlapped beam is passed through a polarizer before reaching the camera. The phase difference accumulated between the orthogonally polarized beams renders high-contrast images^67^.

#### QPM

The basic concept of QPM is to generate an interferogram which has information of the sample encoded in it and from this interferogram, the phase map associated with the sample can be retrieved computationally^66^. The difference with other interferometric techniques is that QPM provides a quantitative measure of the phase associated with the sample. The introduction of a reference beam helps to decouple the phase map associated with the sample from the intensity image. Thus, an interferogram is generated by overlapping the reference beam with the object beam. Now based on the angle between the reference and object beams, QPM systems can be classified into on-axis and off-axis geometries. The so generated interferogram at the camera plane is described as follows.

8 $${I}_{QPM}(\overrightarrow{r})=| {u}_{ref}(\overrightarrow{r}){| }^{2}+| {u}_{obj}(\overrightarrow{r}){| }^{2}+2| {u}_{ref}(\overrightarrow{r})| | {u}_{obj}(\overrightarrow{r})| \cos (\Delta \phi (\overrightarrow{r}))$$

The phase map associated with the sample can be retrieved from Eq. (8) from a single frame via Fourier transform technique^68^ or from multiple frames via phase-shifting algorithms^69^. The choice of the reconstruction algorithm depends on the application. For e.g., if the highest temporal resolution is desired, then the single-shot Fourier transform method of reconstruction is useful, whereas if the highest system resolution is desired, then the phase-shifting method is useful. QPM has been used to obtain quantitative information of gold and dielectric nanoparticles^70^, sub-100 nm liposomes^71^, analysis of sperm cells^72^, study neuronal network activity^73^, cell biology^12,66,74,75^ etc. A comprehensive review on QPM and its applications in biomedicine may be found in refs. ^76,77^.

QPM gives integrated phase information about the scattering sample, i.e., 3D phase information about the sample is not retrieved. To retrieve 3D phase information, the sample may be illuminated under various angles and the scattered far-field recorded^16^. Now to retrieve the 3D quantitative phase information about the sample, multiple 2D holograms as a function of the illumination angle can be utilized to computationally reconstruct quantitative 3D phase map of samples^78^. The penetration depth of these techniques are limited by multiple scattering events and an increase in the penetration depth, <3 mm, can be achieved by resorting to OCT.

#### iSCAT

The working principle of iSCAT is similar to QPM. An object beam, i.e., light scattered off the sample, interferes with reflected light from the sample-glass interface, in an on-axis configuration^65^. The main strength of iSCAT lies in the detection of weakly scattering specimens like viruses or proteins^79^. Since these specimens are smaller than the wavelength of the illuminating light, they may be regarded as Rayleigh scatterers. It means that the strength of the scattered field varies as *a*^6^, for spherical particles with radius *a*. For particles like proteins, the term $| {u}_{obj}(\overrightarrow{r}){| }^{2}$ in Eq. (8) may be neglected. This implies that dark-field detection of such specimens will be challenging. However, in iSCAT the cross-term in Eq. (8) implies that the detected intensity scales linearly with the volume of the scatterer, i.e., *I*_*iSCAT*_ ∝ *a*^3^. Therefore, the contrast generated in the iSCAT image can be written as $\frac{2| {u}_{obj}| }{{u}_{ref}}$. Thus, interference between the scattered and reference fields helps to enhance the sensitivity. Since such weak scattered signals are to be detected, any background signal is minimized in iSCAT by subtracting or dividing images with and without the specimen under investigation. The result is that virus, single proteins are imaged at high temporal resolutions^79,80^. A newer version of iSCAT called COBRI is also gaining popularity for imaging of weakly scattering specimens^81^. COBRI is an abbreviated form for coherent bright field microscopy where a laser is used in bright field configuration. The difference in COBRI is that the reference beam interferes with forward scattered light from the sample whereas in iSCAT it is with the backward scattered light.

**Fig. 2:**
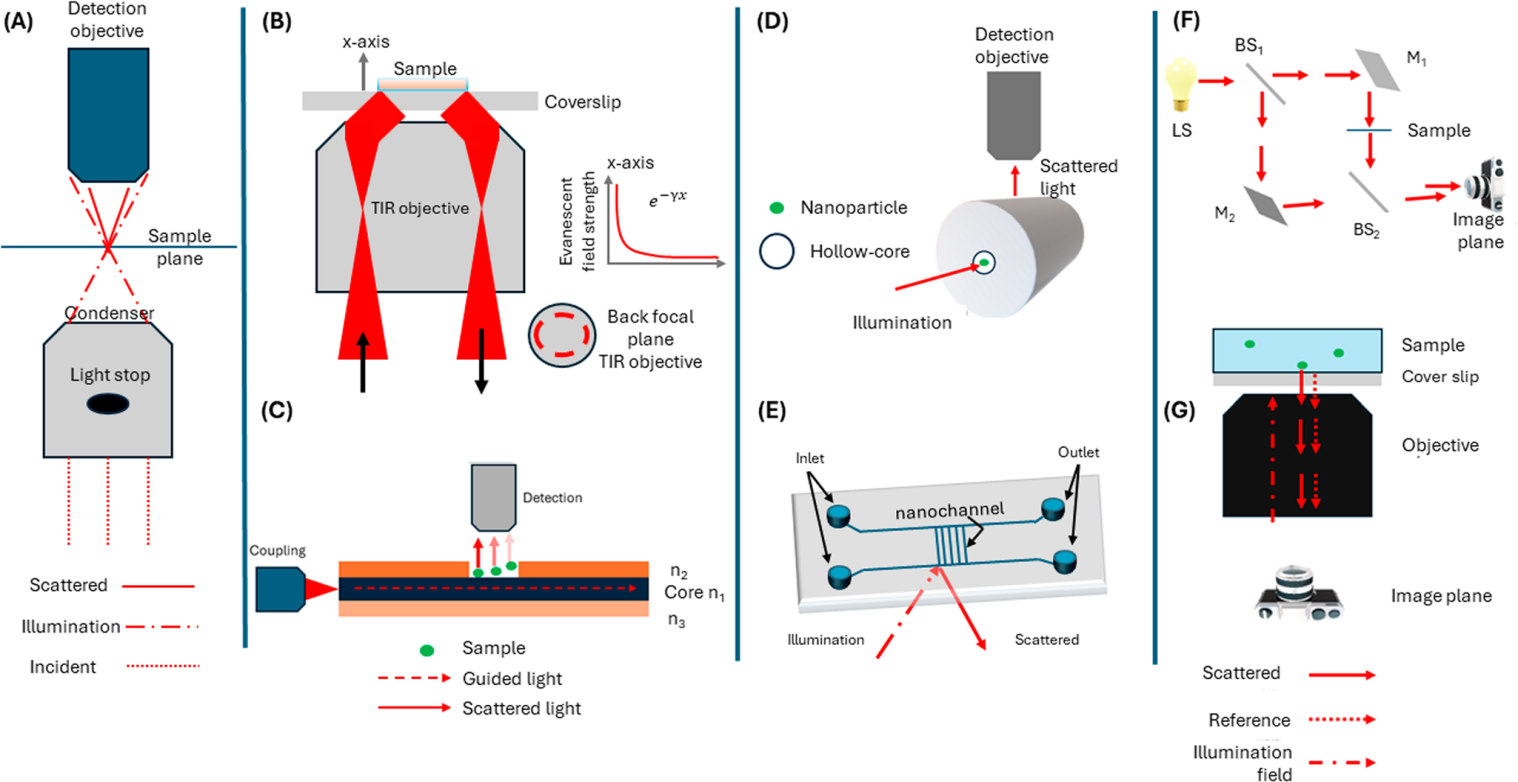
Schematic diagram of certain high-contrast optical imaging techniques. **A** Conventional dark-field: an incident light beam is made to illuminate the sample at oblique angles via a high NA condenser lens that hosts an opaque light stop. The hollow cone of light gets scattered off the sample and only the scattered fields get collected by a lower NA detection objective. **B** Objective-based TIR: an incident beam along the positive *x*-axis is typically focused close to the edge of the back-focal plane of a high NA TIR objective. The sample is enclosed in an appropriate mounting media and mounted on a coverslip. Collimated light that exits the front focal plane can undergo TIR at the coverslip-sample interface. This causes the totally reflected light beam to trace back its path along the negative *x*-axis through the TIR objective. An exponentially decaying wave, called evanescent wave, illuminates the sample. The field strength drops off exponentially, $\exp (-\gamma x)$, thereby permitting imaging of only those structures that are close to the coverslip. To suppress speckle noise and to probe the sample spectrum isotropic, the focused beam is typically made to scan 2*π* radians at the back focal plane. **C** Dielectric waveguide-based : using an objective MO_1_, light is focused into a higher-index (*n*_1_) core of a waveguide, sandwiched between lower-index claddings (*n*_1_ > *n*_2_ and *n*_3_), which gets guided along its length due to TIR. Simultaenously an evanescent field gets setup along the length and interacts with the sample placed on the core-cladding interface. The scattered field off the sample is collected using another detection objective MO_2_, i.e., decoupled illumination and detection scheme allows the scattered fields to be imaged with variable field-of-views. **D** Simplified hollow-core fiber scheme: light gets guided inside the hollow-core and interacts with the particles freely diffusing in it. The scattered far-field radiation is collected using a decoupled detection scheme. **E** Nanofluidic scattering microscopy: The hollow channel spans tens to few hundred nanometers and helps keep the diffusing particle within the focal volume of the imaging objective. An oblique beam illuminates the sample and only the scattered field off the particles are collected^60^. **F** Digital Holography: light from a light source (LS) is split into two arms using a beam splitter (BS_1_). One arm hosts the mirror M_1_ that directs the light onto the sample and the other arm hosts the mirror M_2_ that acts as a reference beam. The object and reference beams are made to overlap at the camera plane via a beam splitter (BS_2_). The camera records a digital hologram with quantitative information of the sample encoded in it. **G** iSCAT scheme: an incident field illuminates the sample mounted on a coverslip. Reflection from the coverslip-water interface and scattered field off the sample interferes at the image plane to record an on-axis hologram.

In conclusion, the pursuit of both dark-field and interferometric techniques is to image even weakly scattering specimens with high contrast^63,66,82,83^. Figure 2 provides a schematic representation of some of the state-of-the-art high-contrast techniques. However, the importance of coherence of light is almost underplayed despite being of paramount importance for both high-contrast and high-resolution techniques. So what is coherence? Coherence can be defined as a measure of statistical similarity of the interfering beams^84^. To elucidate this concept of coherence, the following simplified example is provided. Assume two monochromatic time-dependent scalar fields ${u}_{1}(\overrightarrow{r},t)=a\cos ({\phi }_{1}(\overrightarrow{r},t)-\omega t)$ and ${u}_{2}(\overrightarrow{r},t)=a\cos ({\phi }_{2}(\overrightarrow{r},t)-\omega t)$ overlapping in space and time, where *a* is the amplitude of the fields, *ϕ* is the time dependent phase, *ω* is the temporal frequency and *t* is the time. The intensity registered by any available optical detector today will record a time-averaged quantity, < *I* > _*t*_, due to the fast oscillations of the field. Then the cross-term of the time-averaged intensity is $< {I}_{12}(\overrightarrow{r},t){\ > \ }_{\!\!\!t}={a}^{2} < \cos ({\phi }_{1}(\overrightarrow{r},t)-{\phi }_{2}(\overrightarrow{r},t)-\delta ) >$, where *δ* is the path-length difference between the fields at location $\overrightarrow{r}$. It is interesting to note that if the phases of fields are correlated, then the spatial modulation in intensity will be $\cos ({\phi }_{1}(\overrightarrow{r})-{\phi }_{2}(\overrightarrow{r})-\delta )$. However, if the phases of the two fields fluctuate randomly, then the argument of the cosine function will be a function of time and hence, time averaging will force the cosine term to zero. As a result, no interference pattern will be observed by the camera^84^. This is the scenario in fluorescence microscopy, where the fluorescent molecules can be assumed to emit incoherently, i.e., the emissions of the molecules over time is uncorrelated. The physical interpretation is that within the integration time of the camera, several momentary spatially distinct interference patterns are generated, which get averaged over the integration time of the camera. So colloquially it can be stated that coherence lies in the eyes of the beholder, i.e., depends on the integration time of the camera. This averaging of various interference patterns can also be understood by invoking the Central Limit Theorem, which states that the sum of ‘N’uncorrelated speckle patterns on an intensity basis will help suppress the contrast of the speckles by 1/$\sqrt{N}$^85^. Thus, Tables 1 and 2 highlight the image formation processes and the typical light sources used some of the state-of-the-art techniques.

**Table 1:** High-contrast dark-field techniques

Sl. no.	Technique	Remarks	Applications
1	Ultramicroscope (Dark-field)	(i) Decoupled illumination and detection paths (ii) Advancement in optics and tissue clearing protocols has popularized the concept of ultramicroscope to label-free light sheet microscopy (iii) Typical light sources used now are lasers and LEDs	Nanopartilce detection^147^ Cytometry^148^ Volumetric imaging^23^
2	Prism-based (Dark-field)	(i) Near-field illumination (ii) Decoupled illumination and detection paths (iii) Typical light source used is laser	Protein interactions in real time^149^ Applications in surface plasmon imaging^150^
3	Objective-based (Dark-field)	(i) Near-field illumination (ii) Limited field-of-view (iii) Typical light source used is laser	Dynamics of molecular motors^32,151^ Virus detection and tracking^152^ Nanoparticle tracking^153^
4	Optical fiber/waveguide (Dark-field)	(i) Opto-fluidic and hollow-core fiber geometry permits confinement of biological sample, thereby permitting better light-matter interaction over longer periods. Preferable for sparse samples due to coherent noise (ii) Dielectric waveguide geometry provides near-field illumination to sample placed on its core-cladding interface (iii) Decoupled illumination and detection (iv) Index-matched geometry or incoherent summation of multiple modes help improve signal to noise ratio. (v) Typical light sources used include lasers and LEDs	Nanoparticle detection, characterization^42^ Single virus tracking^59^ Live cell dynamics^43^

**Table 2:** High-contrast interferometric techniques

Sl. no.	Technique	Remarks	Applications
1	Phase-contrast (Interferometry)	(i) Introduction of phase plate to introduce phase delay between the unscatterd and scattered waves off the sample (ii) Conventional PCM is qualitative (iii) Typical light source used is white light (iv) Spatial light interference microscopy (SLIM) combines the concept of PCM and holography to generate quantitative phase maps	Cancer diagnosis^154^ Cell segmentation and tracking^155^ Cell biology^156^
2	Differential interference contrast (Interferometry)	(i) Lateral shear introduced between interfering beams (ii) Conventional DIC is qualitative (iii) Typical light source used is white light (iv) Gradient light interference microscopy (GLIM) combines the concept of DIC and holography to generate quantitative phase maps	3D bioimaging of cells and embryos^157–159^
3	Digital Holography (Interferometry)	(i) Interference between sample and reference beams leads to interferogram with sample information encoded in it (ii) Computational post-processing to retrieve phase information (iii) Typical light sources used include white light, LED, laser and pseudo-thermal	Cell biology^66^ Cancer research^160^ Nanoparticle characterization^70,71^
4	iSCAT (Interferometry)	(i) Weak scattered light from the sample is detected via interference with a reflected light from the coverslip (ii) Well stabilized setup and temporal background subtraction helps detect weak scattering. (iii) Typical light sources used include laser, LED etc. Requires coherence of the light source to be large enough to ensure interference between the scattered and reference fields.	Protein dynamics^161^ Virus detection and analysis^162^

## Methods to combat poor resolution

In this section, a few of the commonly employed high-resolution label-free techniques is discussed, with focus on linear label-free super-resolved techniques.

### Category 1: perfect lens

As explained in Section “Loss in spatial resolution”, the loss of high spatial frequency components of the scattered light leads to diffraction-limited resolution. Therefore, one of the pressing questions often asked is if a lens can be designed which is capable of creating a perfect image without any blurring. Based on the existing knowledge of diffraction, the natural solution is to design a lens that is capable of transmitting not only the propagating waves but also the evanescently decaying components that have the sub-wavelength information encoded in them. However, this natural solution demands certain unnatural material properties for the lens material. In 2000, Sir John Pendry extended the idea of a lens made of negative refractive index material (NRM) proposed by Prof. Veselago^86^. Negative material parameters are typically observed in artificial materials by engineering their resonant response^87^. An important conclusion of Prof. Victor Veselago was that if a material has both its permittivity (*ϵ*_*m*_) and permeability (*μ*_*m*_) simultaneously negative, then causality demands that the negative sign be chosen for the refractive index, i.e., $n=-\sqrt{{\epsilon }_{m}{\mu }_{m}}$. This negative value of the refractive index has many counter-intuitive physical consequences, and the concept of perfect lens is one among them.

The choice of negative sign for the refractive index can be understood by considering light-matter interaction using the Lorentz/Lorentz–Drude oscillator model and acknowledging that all materials are dispersive, i.e., the response of the material to an applied field, polarization, is not instantaneous. This behavior is formulated mathematically by the Kramer–Kronig relationship which implies that the material parameters are complex functions of frequency. Being a complex function implies that materials are lossy, where the imaginary part accounts for the losses in the medium. Representing the complex permittivity and permeability as *ϵ*_*m*_ = *ϵ*_1_ + *iϵ*_2_ and *μ*_*m*_ = *μ*_1_ + *iμ*_2_ respectively, the complex refractive index can be shown to be as follows in the limit of small absorption.

9 $${n}_{m}\approx \pm \sqrt{{\epsilon }_{1}{\mu }_{1}}\left[1+\frac{i}{2}\left(\frac{{\epsilon }_{2}}{{\epsilon }_{1}}+\frac{{\mu }_{2}}{{\mu }_{1}}\right)\right]$$

A plane wave incident from vacuum on such a medium upon propagation must suffers attenuation. Therefore, the negative root in Eq. (9) is chosen to represent a physically valid solution. Now if *ϵ*_*m*_ = −1 and *μ*_*m*_ = −1 for a flat slab of thickness ‘*d*’, Pendry showed that the flat slab can work as a perfect lens. Such a material is impedance matched with vacuum and therefore, suffers no reflection. The transfer function of the flat piece of slab is calculated and shown for propagating and evanescent waves. The propagation of light in the flat slab cancels the phase accumulated by the propagating waves (low frequency components) and amplifies the evanescent waves (high frequency components) exponentially to cancel their net amplitude change. As a result, a perfect image in principle can be formed at a distance ‘2*d*’can be formed of a point source placed ‘*d*/2’units in front of the lens. An exhaustive review on this topic is in ref. ^88^.

### Category 2: time reversal

The basic problem addressed by employing the concept of time reversal is focusing of light beyond the diffraction limit. Time reversal arises because Maxwell equations obey time reversal symmetry. To understand the concept of time reversal the following simplified example is considered. A converging spherical wave, $\exp (-ikr-i\omega t)/r$, is propagating towards the origin *r* = 0. The wave becomes diverging, $\exp (ikr-i\omega t)/r$, after *r* = 0. The total field, neglecting the time dependence for brevity, will thus be a superposition of the converging and diverging waves, $\exp (ikr)/r-\exp (-ikr)/r=\frac{-2i\sin (kr)}{r}$. The minus sign in front of the diverging wave ensures the avoidance of singularity at the origin and implies destructive interference between the convergent and divergent waves. Hence, due to the superposition of these waves, the field varies as a sinc-function and the full-width half maxima of the field is ~*λ*/2, giving rise to the diffraction limit. If however a point-like absorber is placed at the origin, the diverging wave is no longer present. The field amplitude then tends to infinity close to the origin. In other words, a sub-diffraction-sized focal spot is generated. Explained in terms of spatial frequency components, a converging wave gives rise to evanescent waves in the presence of a sink, thereby leading to the sub-diffraction-sized spot. The generation of evanescent waves for sub-diffraction focusing can also be achieved by controlled focusing of light through scattering medium^89,90^. The experimental realizations of sub-diffraction focusing by employing the time reversal concept have been successfully demonstrated in acoustics^91^, and translating these concepts to visible wavelengths in experiments is actively investigated^90,92^.

### Category 3: super oscillations

As opposed to other methods that utilize evanescent waves for sub-diffraction focusing, superoscillatory functions utilize destructive interference of propagating waves to create a sub-diffraction sized spot^93,94^. They are defined as band-limited functions, i.e., having a compact frequency support, that locally oscillates faster than the maximum Fourier frequency. The term was initially used in optics by Prof. Michael Berry in 1994 and has since kindled the research interests of theorists and experimentalists^95^. This phenomenon of superosicllations is typically explained using the following function in Eq. (10), where *a* > 1 and *N* > > 1.

10 $${F}_{N}(a,x)={\left[\cos \left(\frac{x}{n}\right)+ia\sin \left(\frac{x}{n}\right)\right]}^{N}$$

Around *x* = 0, the function can be approximated using a first-order Taylor expansion and shown to be *F*_*N*_(*a*, *x*) ≈ *exp*(*iax*). A Fourier series expansion of Eq. (10) shows the magnitude of the highest spatial frequency to be one. Hence, it can be seen that *F*_*N*_(*a*, *x*) around *x* = 0 oscillates, at *a* > 1, faster than its highest Fourier component. As a result, a sub-diffraction-sized concentration of optical energy is obtained near *x* = 0. However, the superoscillatory behavior comes at the cost of intense side-bands limiting the FoV. The field is active with various methods being investigated for the generation of superoscillatory beams and its application for sub-diffraction limited microscopy is growing^96^.

### Category 4: oblique illumination for enhanced resolution

A shift in the illumination beam helps transfer the coherently scattered higher spatial frequencies of the grating (i.e., sample) into the pass-band of the objective. Therefore, by sequentially illuminating the sample from various known polar and azimuthal angles, an isotropic high-resolution image may be generated by correctly stitching together the captured diffraction-limited images of the sample, $F(\overrightarrow{k}-{\overrightarrow{k}}_{i})$. This concept of achieving higher resolution is achieved via different experimental configurations, a few of them are mentioned below.

#### Fourier ptychography

The word ptycho means ‘fold’ and Fourier refers to the reconstruction of the high-resolution image using the various low-resolution images in the spatial frequency domain, thereby giving rise to the name Fourier Ptychography (FP). FP was first experimentally demonstrated by Zheng et al. in 2013^97^. It is a computational technique that utilizes multiple-intensity images to approximate the absorption and phase of the sample. The main selling point of the technique is high-resolution images over large FoV, i.e., increased space bandwidth product (SBP) of the system. Typically in FP, a flat light emitting diode (LED) array is used as the light source to illuminate the sample from various angles which also helps in coherent noise suppression^98^. To understand resolution gain arising from such a light source, a good approximation is to deem that a spatially coherent field illuminates the sample. Therefore, for a circular aperture, the coherent transfer function will have a cut-off frequency *k*_*c*_ = 2*πNA*_*det*_/*λ*. It means that using an on-axis LED, the highest spatial frequency admissible into the system is *k*_*c*_. To understand this, the concept of Fourier optics is employed. Assuming a thin one-dimensional sample *s*(*x*) illuminated by a plane-wave *exp*(*ik*_*i*_*x*), the Fourier spectrum of the scattered field can be approximated as $\hat{s}(k-{k}_{i})$^99^. Now what determines the magnitude of the wavevector *k*_*i*_? For a fixed wavelength, it is determined by the angle of illumination for that particular experiment. That is, for an off-axis LED that is used to illuminate the sample

11 $${k}_{i}={k}_{\max }=2\pi {n}_{ill}\sin {\theta }_{\max }/\lambda$$

where *n*_*ill*_ is the medium refractive index between the light source and the sample and *θ*_*max*_ is the maximum illumination angle. Hence, by sequentially illuminating the different LEDs in the array, the final spectrum within [− *k*_*c*_−*k*_*max*_, *k*_*c*_+*k*_*max*_] can be collected. The collected image spectra are then linearly combined and Fourier transformed back to real space to generate the reconstructed image. Translated to resolution in real space, the best achievable resolution of such a system is *NA*_*max*_ = *NA*_*ill*_ + *NA*_*det*_^100^. An even larger magnitude of the wavevector can be achieved, for e.g., by illuminating the sample using an optical waveguide^50^, i.e., *k*_*max*_ = 2*πn*_*eff*_/*λ*.

#### Synthetic aperture microscopy

Like FP, the necessity to develop a synthetic aperture optical microscope (SAM) is attributed to (a) a larger working distance with no degradation in spatial resolution, (b) an improved depth of field with no degradation in spatial resolution and (c) an increased SBP of the system^101^. SBP is a dimensionless quantity and is typically calculated as the product of the imaging system’s FoV and spatial frequency coverage (bandwidth), i.e., high resolution over large FoV^102^. The major difference between FP and SAM is that in SAM the phase recovery of the sample is performed via interferometry whereas in FP, multiple images with a certain amount of redundancy helps in determining the unknown sample phase. The concepts of SAM has been successfully translated to experiments to generate high-resolution images of biological specimens^103^.

#### High resolution via evanescent wave illumination

Evanescent wave illumination helps capture the highest accessible spatial frequencies of the sample using an oblique illumination scheme^104^. Typically coherent light sources like lasers are used in TIR geometry to generate an evanescent wave illumination scheme. And two schemes gaining popularity for achieving an evanescent wave illumination is through the use of high NA objectives or through waveguides^43,50^. Rotating Coherent Scattering microscopy (ROCS) uses a high NA TIR objective to generate an evanescent field at the glass bioloigcal sample interface^105^. As a result, only those sections of the sample which are within the penetration depth of the evanescent field scatter light which is collected by the same objective and relayed onto a camera. Despite the limited penetration depth of the evanescent field, the usage of a coherent light source degrades the image quality due to the generation of speckles. Hence, speckle suppression via averaging several different speckle patterns within the integration time of the camera is performed to generate the final high contrast high-resolution image.

A similar scheme is also employed in waveguides where evanescent wave at the core-cladding interface of the waveguide gets scattered into the far-field carrying the very high spatial frequencies of the sample. Speckle suppression in waveguide geometries have been achieved by exciting multiple modes in the waveguide and averaging the different speckles generated due to the different modes within the integration time of the camera^43^. An alternate way employed in waveguides to suppress speckle noise is through the usage of index-matched core-cladding geometry^42^.

### Category 5: fluorescence-based super-resolution algorithms in label-free regime

Intrinsic autofluorescence in biological specimens can mimic the photokinetics of fluorescent molecules, i.e., the autofluorescent emission is incoherent. Hence, concepts of linear structured illumination microscopy (SIM), single-molecule localization, stimulated emission depletion etc. employed in fluorescence microscopy, can be applied to certain label-free samples to achieve label-free super-resolution^44,106,107^.

Fluorescence-based algorithms have also been applied for coherently scattering specimens^43,108–110^. Though resolution below 100 nm has been demonstrated, these techniques are still diffraction-limited as per the Abbe limit, i.e., the best achievable resolution is given by Eq. (3). It is because application of fluorescence-based algorithms, like SIM^14,111^ or intensity-fluctuation-based techniques^112^ does not provide any gain in resolution over the Abbe limit but may improve the contrast of the final image^43,108,109^. Such high resolutions have been achieved via use of shorter wavelengths or high-index materials, as in hyperbolic material enhanced scattering (HMES) nanoscopy, and application of fluorescence-based algorithms does not give any resolution gain over the Abbe limit^108,109^.

Recently, a technique called EPSLON, Evanescently decaying Photoluminescence enables Label-free Optical Nanoscopy, has been demonstrated where the intrinsic autofluorescence in Si_3_N_4_ waveguide is used for near-field illumination of unlabeled samples. The near-field illumination is to ensure that correlations between different scattering points do not arise because the correlation function satisfies the wave equation^84^. Then the scattered light off the sample mimics fluorescence emission, i.e., incoherent emission thereby permitting the application of fluorescence-based SIM and intensity-fluctuation techniques for label-free microscopy to achieve super-resolution^113^. Label-free super-resolved images of gold, polystyrened nanoparticles, extra-cellular vesicles and biological tissues with sub-150 nm mean resolution is demonstrated.

### Category 6: optical illumination and electron beam detection

Optical Near-Field Electron Microscopy (ONEM) is a recent technique that utilises the non-invasive nature of optical waves for sample illumination and the high resolution associated with electron waves for detection of the scattered field^114^. The near-field scattering information, i.e., both propagating and evanescently decaying waves arising from the unlabeled sample is directed onto a photocathode placed in close proximity to the sample. This scattered light of the sample containing near-field information gives rise to a spatially varying electron flux due to photoelectric effect in the photocathode. These emitted photoelectrons are then imaged using a low energy electron microscope to generate a high resolution image, ~5 nm, of the sample.

**Fig. 3:**
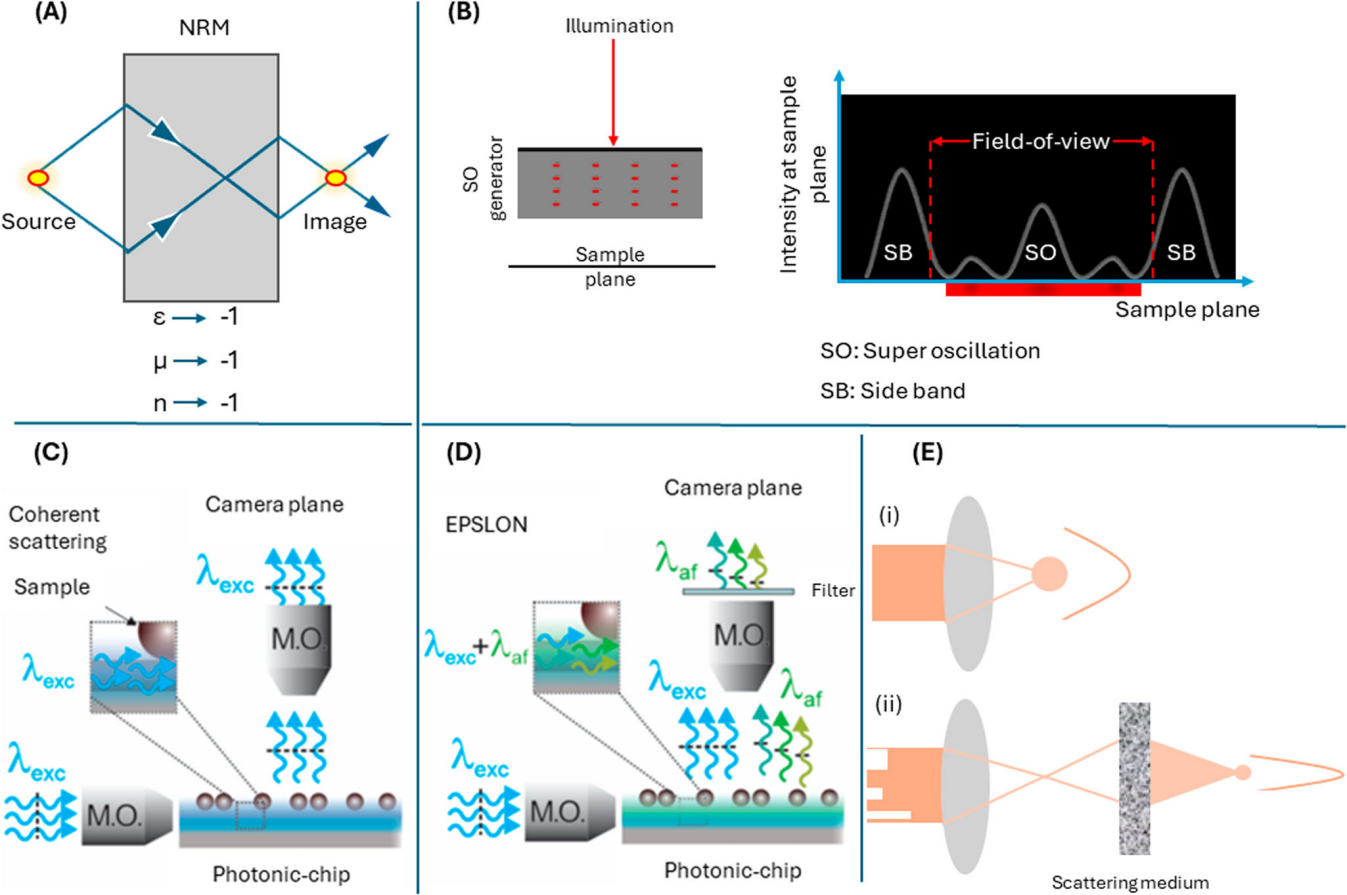
Schematic diagram of certain high-resolution optical imaging techniques. **A** Negative refraction: A negative refractive index material (NRM) can be used to generate a perfect image, i.e., all the Fourier components scattered off an object can be focused. Typically using a thin lens made of silver, a super-resolved image can be generated. **B** Super-resolution via super-oscillatory (SO) spots: One way of achieving SO spots is via sending light through an array of quasi-periodic nanoholes which is referred to as the SO generator. The generation of SO spot is accompanied with the generation of intense side-lobes, which limits the field-of-view for sub-diffraction imaging. **C** Coherent scattering: Oblique illumination helps transfer the higher-spatial frequencies of the sample into the pass-band of the detection objective. The highest transferable Fourier components of the object depend on the magnitude of the illumination wavevector. For e.g., optical waveguides can help achieve a high wavevector due to the high refractive-index of the core guiding the light. Using photonic-chips, near-field illumination, *λ*_*exc*_, of unlabeled samples causes the higher Fourier components of the sample to be transferred to the passband of a detection objective which is typically employed in an orthogonal geometry. Computational post-processing can help to restore the captured Fourier spectrum of the sample to appropriate locations in the Fourier space, and a high-resolution diffraction-limited image can be generated. **D** EPSLON: Here the photoluminescence of Si_3_N_4_ waveguides is utilized for near-field illumination of unlabeled samples. The coherent excitation light and the incoherent photoluminescence are both scattered into the far-field by the unlabeled sample. Using appropriate spectral filters, the coherent light is cut-off and only the incoherent light is collected by the orthogonally placed detection objective and allowed to reach the camera. **E** Time Reversal: Light focused using a conventional lens generates a diffraction-limit sized spot. However, by spatially modulating the wavefront of an incident light beam passing through a scattering medium, a sub-diffraction sized focal spot can be generated.

In conclusion, a number of different concepts have emerged to tackle the issue of poor spatial resolution and Fig. 3 provides a schematic illustration of some of these techniques. Capturing and restoring the evanescent waves to generate super-resolved images as in perfect lens, creating sub-diffraction sized spot as in super-oscillations, highly-oblique illuminations to capture the higher spatial frequencies of the sample as in ROCS, novel materials functioning as the condenser as in HMES nanoscopy, manipulating the coherence of the scattered light to achieve super-resolution via fluorescence-based super-resolution algorithms as in EPSLON are certain techniques to help push the resolution below 200 nm. Table 3 provides a gist of techniques such as perfect lens, time reversal, super-oscillations and ONEM while Table 4 provies a gist of techniques that have demonstrated high-resolution imaging on biological samples.

**Table 3:** High-resolution techniques

Sl. no.	Technique	Remarks	Applications
1	Perfect lens (Super-resolution)	(i) True super-resolution in principle by restoring evanescent waves emanating from the sample in the image plane (ii) Difficulty in engineering negative refractive index due to fabrication challenges (iii) Typical light source used is laser and requires resonant operation of the material. Therefore, bandwidth limited operation	Proof-of-concept results demonstrated, mostly on non-biological samples^163,164^
2	Time reversal (Super-resolution)	(i) Primary aim is to generate sub-diffraction focus (ii) Presence of an absorber introduces evanescent components needed for sub-diffraction focusing (iii) Typical light source used is laser	Sub-diffraction focusing for biological imaging is actively pursued^90^
3	Super-oscillations (Super-resolution)	(i) Functions can oscillate locally faster than its highest Fourier component (ii) Sub-diffraction spot in the far-field (iii) Limited field-of-view due to intense side lobes (iv) Typical light source used is laser	Emerging field with proof-of-concept results on fluorescent biological samples^165^
4	ONEM: Optical illumination and electron beam detection (Super-resolution)	(i) Optical excitation (ii) Near-field sample information converted to photoelectrons (iii) Low energy electron microscope based detection	Emerging field^114^

**Table 4:** High-resolution techniques on biological samples

Sl. no.	Technique	Remarks	Applications
1	Oblique illumination (Abbe limited)	(i) Oblique illumination in ROCS helps probe finer details of the specimen. Oblique illumination and computational post-processing as in as in Fourier ptychography or synthetic aperture microscopy helps increase passband of the object spectrum (ii) FP and SAM generate amplitude and phase information of the sample and therefore, quantitative (iii) Typical light source used is laser or LED	Live cell biology^166,167^ Tomography^103,168,169^ Pathology^170^
2	Fluorescence-based super-resolution algorithms to coherently scattering specimens (Abbe limited)	(i) Non-uniform illumination helps induce intensity fluctuations (ii) These fluctuations arise due to phase of the illuminating field, which prevents gaining resolution over the Abbe limit via fluorescence-based algorithms (iii) Typical light source used is laser	Nanoparticles^43,110^ Cell biology^171^
3	Fluorescence-based super-resolution algorithms to autofluorescent samples (Super-resolution)	(i) Incoherent autofluorescent emission helps circumventing the Abbe limit (ii) Concept of stimulated emission depletion (STED) and single photon localization microscopy applied to autofluorescent samples (iii) Imaging restricted to autofluorescent samples (iv) Typical light source used is laser	Super-resolved images of thylakoid membrane^106^ Super-resolved features of chromatin^107^
4	EPSLON: Fluorescence-based super-resolution algorithms on to incoherently scattering unlabeled samples (Super-resolution)	(i) Photoluminescence in Si_3_N_4_ waveguides for near-field illumination of unlabeled samples (ii) Concept of structured illumination microscopy (SIM) and intensity-fluctuation applied to unlabeled incoherently scattering samples for circumventing the Abbe limit (iii) Imaging restricted to near-field (iv) Typical light source used is laser	Super-resolved images of nanoparticles and biological tissues^113^

Though, in this article the onus has been primarily on explaining the physical mechanism behind the image formation process associated with various techniques, it is imperative to highlight a few other label-free techniques as well due to its applications in life sciences. Polarization microscopy relies on birefringence or optical anisotropy in samples^115^ like mitotic spindles^116^, oocytes^117^ etc. to generate contrast and aid in diagnosis. The transversal spatial resolution of this technique is hundreds of nanometers and the imaging depth is few micrometers.

Now, non-linear light-matter interactions like second or third-harmonic generation utilizes the second or third-order endogenous susceptibility of samples to generate the contrast. With a transversal resolution of hundreds of nanometers and imaging depth of hundreds of micrometers, these non-linear optical imaging techniques help extensively in disease diagnosis^118–120^. For deep tissue imaging, OCT^121^ is one of the more popular techniques for clinical applications. Image contrast is generated via a low coherent interferometer, i.e., only when the optical path lengths between the sample and reference arms of the interferometer are matched does a signal arise. Some of the clinical applications of OCT include opthamology^122^, dentistry^123^, cardiology^124^ etc. Autofluorescent microscopy utilizes the photoluminescence properties of endogenous flurochromes in biological samples to generate contrast. They can generate spatial resolutions of hundreds of nanometers and image upto a few hundred micrometers deep if the sample is not highly scattering. Their applications include cell classification^125^, tissue imaging and quantification^126^ etc. Biomedical spectral imaging, i.e., integration of imaging and spectroscopy, also employs the concept of light-matter interaction to generate contrast. The response of matter to light is strongly dependent on the frequency of the illumination field and this property can be used to study physiological status of tissues, understanding of diseases, cellular analysis etc. An extensive review article on this field may be found in refs. ^127,128^. These techniques provide penetration depth of few hundred micrometers with spatial resolution on the range of few to tens or hundreds of micrometers.

An improvement in spatial resolution, down to a few hundred nanometers can be achieved via ultraviolet (UV) light, albeit at the cost of reduced penetration depth due to stronger absorption of UV light by biological samples. Applications of UV microscopy include molecular imaging and hematology analysis^129,130^, image nanopores like fenestrations in liver sinusoidal endothelial cells^131^, high-resolution imaging utlizing the autofluorescence of yeast cells^44^ etc. However, exposure to UV can be detrimental for biological samples^132^ and the availability of imaging components at UV wavelengths is limited. To gain complementary information about the sample like morphology, biochemical properties etc., multi-modal imaging is becoming popular^133–135^ and finding applications in cell culture research, diagnosis etc. Label-free characterization of biological nanoparticles such as single proteins^136^, extracellular vesicles (EVs), liposomes etc., has been an active area of research over the last two decades. This is primarily due to possibilities for early diagnosis, therapeutics and drug research^137,138^. Due to the limited refractive index contrast and size, both imaging and characterizing these nanosystems is challenging^83^. Electron microscopy can help study such nanosystems, however, the time-consuming sample preparation protocol can affect the morphology of these systems and can also introduce artifacts. Near-field atomic force microscopy can also be used for studying the morphology of such nanosystems. But the near-field probing and binding of sample on extremely flat substrates introduces experimental challenges. Also, light scattering techniques such as DLS and multi-angle light scattering suffer from the non-linear dependence of the scattering intensity on the particle radius and therefore, imposes detection challenges. Consequently, there has been a growing interest in developing alternate optical techniques, for e.g., optical waveguides^139^, interferometric microscopic techniques^140^, photothermal microscopy etc. to detect and characterize such weakly scattering specimens. The field is active with various research groups across the world trying to push to the label-free detection limit via different techniques^141–143^.

## Discussion and outlook

Despite being minimally invasive, the use of label-free microscope for bio-imaging has suffered setbacks due to limited spatial resolution, poor contrast, lack of specificity and limited penetration depth. This prompted new research directions that utilize the physical properties of electrons, or X-rays, or acoustic waves and their interaction with matter to circumvent these challenges. But the necessity to study life with minimal invasion and overcome these challenges meant developing new concepts and techniques to use visible light itself to shed light on the nature of life. Among them, fluorescence microscopy has enabled circumventing many of the limitations associated with label-free microscopy. Despite its success, the use of exogenous labels meant additional sample preparation steps, limited photon budget at the camera plane, photo-bleaching, photo-toxicity etc. Hence, the research field of label-free microscopy still has offerings worth pursuing. And as a result, over the past few decades, the development of label-free imaging can be classified into either addressing the problem of poor contrast, arising due to weak scattering associated with phase objects or speckle noise due to multiple scattering associated with heterogeneous or thick samples, and limited spatial resolution.

As outlined in this article, there exist several label-free microscopy techniques. The choice of a particular technique is typically based on the intended application. The central challenge associated with imaging of nanometer-sized biological particles is shot-noise limited detection, i.e., picking minuscule scattering from a nanometer-sized particle in a media with a similar refractive index. As outlined in Section “Methods to combat poor contrast”, the concepts developed to pick these weak signals primarily rely either on interference, or dark-field-like concepts, or/and computational post-processing. To improve the detection sensitivity, many of these techniques use sparse samples, which enables single particle tracking and in minimizing coherent noise, or/and fluidic schemes, to limit the Brownian motion to improve the detected photon budget, surface functionalisation schemes again which also aid in improving the detected photon budget. Dark-field, evanescent field excitation using index-matched waveguides etc allow imaging of only scattered fields of the particles to reach the camera, thus improving the detection sensitivity. While iSCAT uses the concepts of interference to mitigate the influence of the nonlinear dependence of the scattering signal on the size of the scattering particle and computational post-processing. For imaging cells and thin tissue sections, methods such as QPM, DIC and PCM, that use concepts of interference, are explored routinely albeit often with limited spatial resolution. Good contrast and an even higher resolution can be achieved by invoking the concept of interference and oblique illumination scheme as in FP or SAM. Imaging of only scattered fields using a dielectric waveguide-based can also be used for generating high contrast high-resolution images. Such techniques which cater to the imaging of cells and tissues typically manipulate the coherence of the illumination light to mitigate speckle noise at the camera plane. This speckle noise can arise due to spurious reflections in the detection arm of the microscope or due to multiple scattering within the sample. As a result, coherent noise suppression is of paramount importance and these techniques typically use light sources such as white light, LED, lasers passed through rotating diffusers, high-index core multi-moded waveguides etc. In addition to such classical light sources, quantum light sources have been shown to achieve sub-shot noise limited detection and improved spatial resolution and is, therefore, a highly researched area and holds potential^144^.

Now, the field of label-free high-resolution and super-resolution microscopy has been generating a lot of interest since the advent of fluorescence-based super-resolution techniques and the proposal of the concept of perfect lens by Pendry. It is worth stressing here that several techniques have demonstrated sub-200 nm resolution^38,39,103,105,110^. These techniques are still diffraction-limited as per the definition of Abbe. A high spatial resolution is achieved because of the usage of shorter wavelength or higher-index materials which helps in increasing the magnitude of the illumination wave-vector and thereby, in transmitting the higher spatial frequencies of the sample into the pass-band of the objective. Therefore, categorizing these techniques as super-resolution is not valid.

Super-resolution techniques have been demonstrated via NRMs, time reversal, super-oscillatory spots, utilizing intrinsic autofluorescence of biological specimens along with fluorescence-based super-resolution algorithms, utilizing photoluminescence of Si_3_N_4_ waveguides for near-field illumination of unlabeled samples in tandem with fluorescence-based super-resolution algorithms. Some of these super-resolution methods, like EPSLON, have demonstrated sub-150 nm super-resolution on biological samples as well, albeit in TIR mode. The ultimate goal of label-free microscopy would be to achieve atomic-level isotropic resolution and sensitivity. But immediately feasible goals seem to be addressing issues such as lack of specificity and multiple scattering from dense biological sections. Here, there exists the potential of using machine learning tools to develop virtually stained images. The use of virtual classification, segmentation, and staining can bring in specificity and rapid diagnosis^145,146^. The quest to address these issues of poor contrast and resolution need not be merely seen as an engineering or technological challenge to solve problems in life sciences. Rather, it is also to be deemed as a quest to discover new physics of light-matter interaction to unravel the mysteries of the microscopic world. A joint effort between physicists, biologists, mathematicians, computer scientists, engineers and of course public funding will help in further propelling this field of label-free microscopy.

## Data Availability

No datasets were generated or analysed during the current study.
